# Supervisors' perspective on medical thesis projects and dropout rates: survey among thesis supervisors at a large German university hospital

**DOI:** 10.1136/bmjopen-2016-012726

**Published:** 2016-10-14

**Authors:** Elif Can, Felicitas Richter, Ralitsa Valchanova, Marc Dewey

**Affiliations:** 1 Charité Graduate Programme, Charité—Universitätsmedizin Berlin, Germany; 2 Department of Radiology, Charité—Universitätsmedizin Berlin, Germany

**Keywords:** scientific work, medical dissertation, medical research

## Abstract

**Objectives:**

To identify underlying causes for failure of medical thesis projects and the constantly high drop-out rate in Germany from the supervisors' perspective and to compare the results with the students' perspective.

**Setting:**

Cross-sectional survey. Online questionnaire for survey of medical thesis supervisors among the staff of Charité—Universitätsmedizin Berlin, Germany. Published, earlier longitudinal survey among students for comparison.

**Participants:**

1069 thesis supervisors participated.

**Data extraction and synthesis:**

Data are presented using descriptive statistics, and the χ^2^ test served to compare the results among supervisors with the earlier data from the longitudinal survey of doctoral students.

**Primary and secondary outcomes:**

Not applicable. This survey is an observational study.

**Results:**

Of 3653 potential participants, 1069 (29.3%) supervising 3744 doctoral candidates participated in the study. Supervisors considered themselves to be highly motivated and to offer adequate supervision. On the other hand, 87% stated that they did not feel well prepared for thesis supervision. Supervisors gave lack of timeliness of doctoral students and personal differences (p=0.024 and p=0.001) as the main reasons for terminating thesis projects. Doctoral students predominantly mentioned methodological problems and difficult subjects as critical issues (p=0.001 and p<0.001). Specifically, students felt ill prepared for the statistical part of their research—49.5% stated that they never received statistical assistance, whereas 97% of supervisors claimed to help their students with statistical analysis.

**Conclusions:**

The authors found that both thesis supervisors and medical students feel ill prepared for their roles in the process of a medical dissertation. Contradictory reasons for terminating medical thesis projects based on supervisors' and students' self-assessment suggest a lack of communication and true scientific collaboration between supervisors and doctoral students as the major underlying issue that requires resolution.

**Strengths and limitations of this study**

- To the best of our knowledge, this is the first study providing a detailed analysis of the supervisors' perspective on medical thesis projects.
- This survey had a rather low response rate of 29%.
- This was a cross-sectional online survey among medical thesis supervisors of Charité—Universitätsmedizin Berlin, Germany and the results were compared with an earlier longitudinal survey among doctoral candidates. The survey may not be representative of the perspective of medical thesis supervisors elsewhere in Europe.
- The results are based on a cross-sectional survey and do not reflect temporal dynamics.

## Introduction

Thesis projects are an important part of research performed at medical schools in Germany and considerably contribute to publications during and after the completion of medical studies.^1–4^ In the German system, medical students qualifying as physicians are not automatically entitled to the title of doctor of medicine (‘Dr. Med.’ in German) but must earn this title by completing an additional dissertation, often under enormous pressure.^5–10^ As a result, a recent *Nature* Editorial has criticised the German system as being conducive to poor scientific standards.^11^ In 2013, 456 students started doctoral thesis projects at Charité—Universitätsmedizin Berlin in Germany, but at the time of the survey only 79% successfully completed their projects and obtained the title of medical doctor.^12^ Thus, about 20–30% of the doctoral candidates never complete their thesis and drop out.^13^ ^14^ The research carried out by doctoral candidates accounts for a third of all publications of the Charité—Universitätsmedizin Berlin,^3^ ^15^ and this is an important motivation to find out why almost a third of doctoral students cancel their projects. In a previously published work, it was shown that candidates pursuing a clinical thesis are more dissatisfied than their fellow candidates working on experimental theses.^2^ ^3^ ^13^ ^16^ The major reasons for the failure to complete medical theses were lack of support from the thesis supervisor and lack of financial resources.^13^ ^16^ ^17^ Two studies investigating the constantly high dropout rate for thesis projects in Germany from the perspective of doctoral candidates pursuing dissertations identified poor supervision as a major cause of failure.^13^ ^16^ Therefore, the Charité graduate programme was established, offering supervised peer education to improve doctoral candidates' scientific, organisational and technical skills for tackling thesis projects.^13^ ^16^ ^18^ ^19^

A deeper insight into what leads to the termination of thesis projects may help identify reasons for low academic standards and measures that can improve the success of medical thesis projects. Therefore, we conducted an online questionnaire survey among medical thesis supervisors to complement existing data by adding the supervisors' perspective. In a second step, the results of this survey were compared with the results of our earlier survey among medical students at the same university,^13^ ^16^ in order to obtain a comprehensive appraisal of why so many candidates do not complete their medical thesis projects.

## Methods

### Study context

We designed a cross-sectional study including supervisors of medical thesis projects at Charité—Universitätsmedizin Berlin (http://www.charite.de). In order to analyse the dropout rate from supervisors' perspectives, we first describe their position in general, including their motivation, reasons for terminating theses and formal training and preparation for the role of supervisor. In a second step, the results of this survey among thesis supervisors were compared with a longitudinal survey among doctoral candidates published in 2014.^13^ ^16^

Both this longitudinal survey and the current survey among supervisors^13^ ^16^ relied on the participants' self-assessments.^20^ ^21^

### Participants

Potential addressees of our questionnaire were all Charité employees with a university degree qualifying them to supervise a medical thesis. A total of 3653 potential medical thesis supervisors were identified and email addresses extracted from the central information and intranet contact portal of Charité. An email invitation to participate in our survey was sent to all potential medical thesis supervisors in January 2014. Nearly one-third (29.3%) of them participated (1069 of 3653).

The longitudinal survey among doctoral candidates had very similar response rates: 28% participated in 2011 (303 of 1081) and 31% in 2001 (321 of 1036).^13^ ^16^

### Data collection

**Figure 1:**
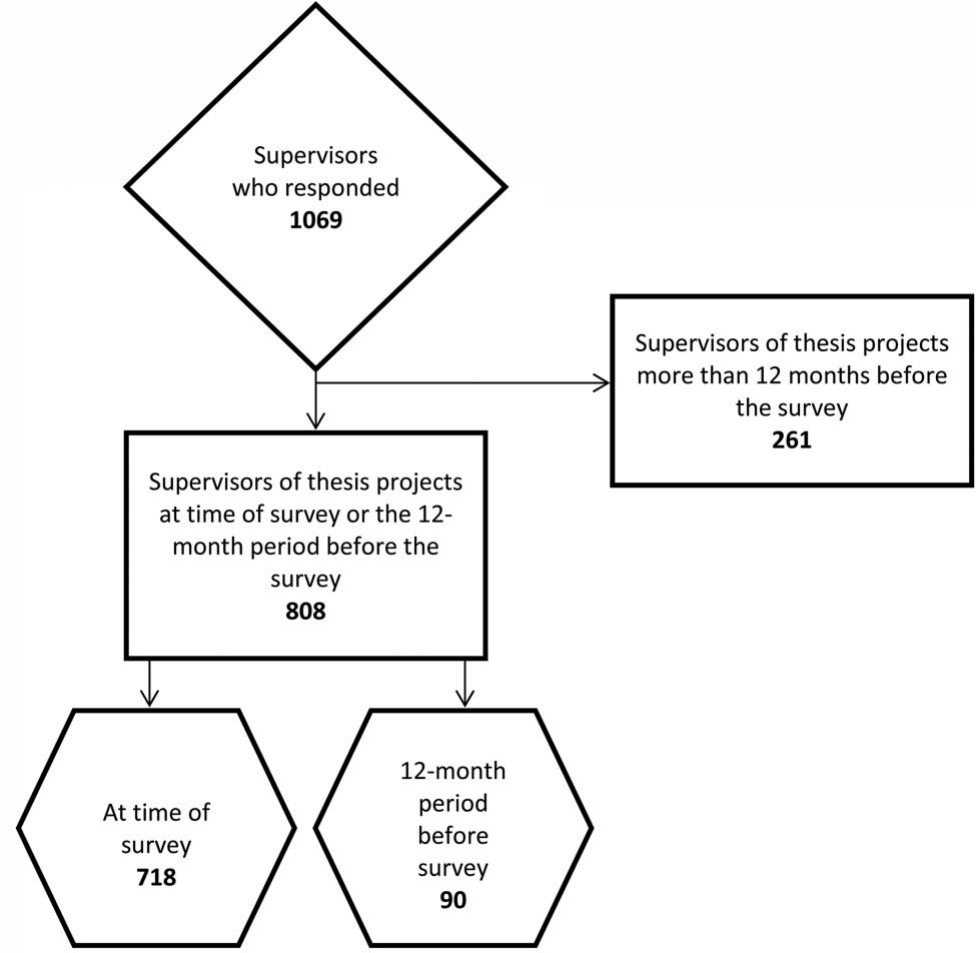
To avoid a recall bias, our analysis is based on those supervisors who supervised thesis projects at the time of the survey or during the 12-month period preceding the survey (n=808). For correct extraction we used decision questions; therefore, the number of respondents is not the same for all questions.

Preparations for the questionnaire survey began in November 2013 by implementing a search tool to identify and invite potential thesis supervisors as described in the preceding section. For the present analysis, we tried to avoid a recall bias by restricting participation in the survey to those who actually supervised a medical thesis during the 12-month period before mailing the questionnaires. For correct extraction we used decision questions. The questions in the survey were logically linked, so that the answer of one question determined which questions to ask next; therefore, the number of participants answering a question is variable (figure 1). Two email reminders were sent by the Dean of Charité (Professor Grüters-Kieslich) and Professor Dewey during the survey, which was open from the beginning of January 2014 until March 2014.

### Questionnaire

The questionnaire was developed using LimeSurvey (https://www.limesurvey.org/en/) and included a total of 65 questions. In terms of content, the items were defined by using common methods and expert validation.^22–26^ Programming details and the entire questionnaire can be provided on data sharing request. LimeSurvey enables access to questions depending on the answer to previous questions. Use of these intelligent linkages allowed personalisation of the questionnaire by means of decision questions, thereby minimising the number of questions for each responder. This approach was chosen in order not to deter potential participants by the large total number of questions and explains the variation in sample sizes for different items on the questionnaire. The survey was split into 12 sections: general information, supervision situation, support of doctoral candidates, contact with candidates, graduation procedure, statistical support, candidates dropping out in the past 12 months (due to supervisor and due to candidates), time expenditure, knowledge about the Charité graduate programme (http://www.promotionskolleg.de), suggestions for improving the current situation and personal information. For the present study, we selected those points collating with the results of the survey among students; specifically, these are all questions relating to the premature termination of thesis projects and the items concerning supervisors’ perception of the current situation. The graduate programme was established at the Charité in 2002 as a direct consequence of the general picture that had emerged from our first survey among medical doctoral candidates in 2001, namely that candidates in general considered medical thesis supervision to be inadequate and that the university did not prepare them adequately for doing research.^16^ This voluntary programme offers courses taught by students to assist doctoral candidates in planning and organising dissertation projects and to provide fundamental methodological knowledge such as statistics. This survey included different question types, such as multiple choice, numerical or text input, and took on average 35 min to complete.

### Statistical analysis

Statistical analysis was performed with SPSS, V.18, and R 2.15.1 (http://www.r-project.org). Data are presented using descriptive statistics. The χ^2^ test was used for comparing groups, and p values of 0.05 were considered to indicate statistically significant differences.

## Results

About 75.6% of the respondents (808 of 1069) were supervisors of thesis projects during the 12 months prior to the survey, and 67.2% (718 of 1069) were supervising a thesis at the time of the survey (figure 1).

### Motivation for supervising thesis projects

**Figure 2:**
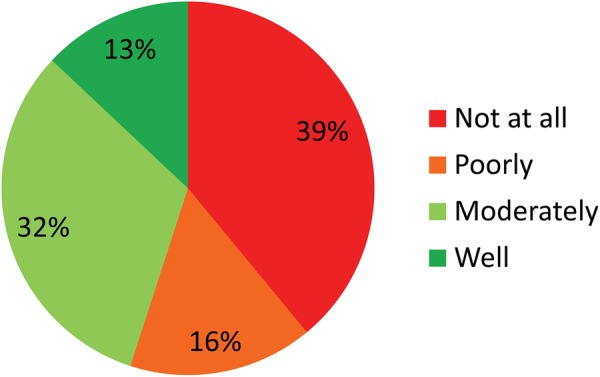
More than half of all respondents feel poorly or not at all prepared for the role of supervising doctoral candidates (n=948) by Charité—Universitätsmedizin Berlin. The period to which this question related was not restricted to the past 12 months.

At the time of the survey, 67.2% of respondents (718 of 1069) were supervising 3744 doctoral candidates (figure 1), corresponding to an average 5.2 doctoral candidates per supervisor. Asked about their motivation for fostering medical thesis projects, most thesis supervisors, 63% (459 of 718), ticked off ‘sharing enthusiasm for their own field’, followed by ‘job satisfaction’ (55%, 395 of 718) and the ‘option of mutual future projects’ (53%, 380 of 718). Promotion of their own scientific career was the motivation for 44% (316 of 718) of supervisors, whereas 37% (266 of 718) considered thesis supervision one of their core responsibilities. Only 13% felt well prepared for supervising medical theses, while 39% reported that they were not at all prepared for this role (figure 2).

### Dropout rate and reasons for dropping out

**Figure 3:**
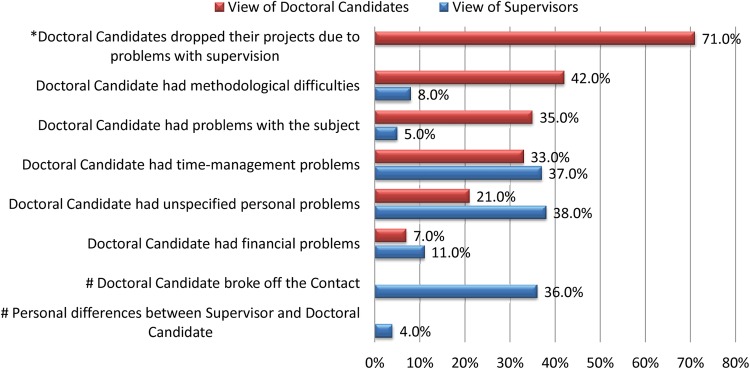
Collated responses given by students, 13 (n=66) and supervisors (n=158); multiple answers were possible. Questions marked with * were only asked in the student survey; questions marked with # were only asked in the supervisor survey. According to supervisors, doctoral candidates most commonly terminated projects because of unspecified personal reasons. In contrast, the most common reasons given by students were difficulties with methodology, including statistical problems.

In our survey 25% of the supervisors (156 of 624) stated that a total of 208 of medical thesis projects were discontinued, while 44% of the supervisors (344 of 782) stated that a total of 598 projects had been completed successfully. Fifty-eight doctoral projects were prematurely ended by 11% of supervisors (47 of 436), while 28% of supervisors (109 of 386) reported a total of 150 thesis projects terminated by the candidates themselves. Supervisors stated that doctoral candidates most commonly terminated projects because of unspecified personal problems or time management problems, or simply failed to show up. In contrast, the reasons given by doctoral candidates in our earlier survey differed greatly (figure 3).^13^ Comparison of the most common reasons for termination of thesis projects given by supervisors and candidates using the χ^2^ test yielded significant differences (table 1).^13^

**Table 1:** Reasons for failure of medical thesis projects according to supervisors versus candidates.

	Candidates (N=98)	Supervisors (N=107)	p Value
Methods	29 (29.8)	9 (8.4)	0.001
Topic	24 (24.5)	6 (5.6)	<0.001
Timelines	23 (23.4)	40 (37.3)	0.024
Personal	14 (14.9)	41 (38.3)	0.001
Finance	7 (7.5)	11 (10.2)	0.281

Values are numbers (percentages).

Reasons for dropout given by thesis supervisors comparing projects terminated by candidates with those terminated by supervisors. About 29.8% of abandoned doctoral projects were terminated by the candidates for methodological reasons. Only 8.4% were discontinued by thesis supervisors for the same reasons. In contrast, 37.3% of thesis projects were ended prematurely by supervisors because of timelines.

Supervisors assumed difficulties with managing timelines and personal differences of students (p=0.024 and p=0.001) as the true reasons for dropout, whereas doctoral candidates more often gave difficulties with research methodology or the thesis subject in general as reasons for terminating the project (p=0.001 and p<0.001). Therefore, we further compared the opinions of thesis supervisors and doctoral candidates about received statistical support. Candidates felt poorly prepared for the statistical part of the project before or during conduct of their research (p=0.019 and p<0.001) with 49.5% stating that they never received statistical support.^13^ In contrast, about 97% of the supervisors believe that they provided statistical help. Only 3.4% of doctoral candidates confirm that they have received support before, during and after practical work on the thesis project (table 2).

**Table 2:** Comparison of candidates' and supervisors' perception of statistical support received/given.

Statistical support	Candidates (N=206)	Supervisors (N=672)	p Value
Never	102 (49.5)	15 (2.2)	<0.001*
Before practical work	24 (11.7)	45 (6.7)	0.019
During practical work	26 (12.6)	238 (35.2)	<0.001*
After practical work	32 (15.5)	69 (10.2)	0.036
Before and during	2 (1.0)	37 (5.5)	0.006*
Before and after	2 (1.0)	27 (4.0)	0.033
During and after	11 (5.3)	105 (15.5)	<0.001*
Before, during and after	7 (3.4)	136 (20.1)	<0.001*

Overall χ²-independence test: p<0.001, pairwise test results are listed in the last column (significant results at a Bonferroni-corrected 95% level of p<0.00625 are marked with *). Almost 50% of doctoral candidates state a lack of statistical support, 13 although approximately 97% of supervisors state to have provided statistical help.

Values are numbers (percentages).

## Discussion

In this cross-sectional study, we investigated the German system for acquiring the academic title of doctor of medicine focusing on the role of supervisors and their views. Following our longitudinal survey among medical doctoral candidates at Charité,^13^ ^16^ the current survey among supervisors was primarily motivated by an interest in finding out why so many candidates do not complete their thesis projects despite the graduate programme existing at Charité since 2002. Both the longitudinal survey among students^13^ ^16^ and the present survey among supervisors were based on self-assessment.^20^ ^21^ While there are problems with self-assessment, this does not degrade our work because we are not concerned with competency, but with the subjective perception of the situation, which affects the behaviour leading to drop-out. Our statistical findings suggest that the drop-out rates are correlated with the respondents' self-assessments. On a more general level, the current survey and our earlier surveys,^13^ ^16^ provide important insights into the situation of junior medical research in Germany in general and draw attention to what needs to be done to adequately prepare medical students before embarking on their first major research project. Taken together, our two earlier surveys among doctoral candidates and the current survey provide an important database for how the system can be improved. The system has recently come under attack through media coverage of prominent cases of plagiarism, which have unveiled an inherent problem of the German system: namely that junior physicians in Germany are under extreme pressure in accomplishing a medical dissertation, often while doing full-time residency training, as succinctly described in a recent *Nature* Editorial.^11^ Critics of the German system claim that this situation is conducive to laxness and low standards of medical dissertations.

For our current cross-sectional survey among thesis supervisors at Charité—Universitätsmedizin Berlin, we identified 3653 potential participants and achieved a response rate of nearly 30%, which is slightly higher than the response rate commonly reported in internet surveys^27^ ^28^ not using a multimodal approach^29^ ^30^ but lower than the response rate normally expected in survey studies (> 65%).^31–33^

Despite the low response rate, there is no problem for data analysis in terms of selection bias, for the following reasons: updating of the intranet always lags behind the high fluctuation of staff. Thus, our email invitation to participate in our survey included both staff who no longer worked at Charité—Universitätsmedizin Berlin (eg, guest scientists, emeritus professors) at the time of the survey and staff who had just started recently and could not yet have become supervisors of doctoral projects. Moreover, it is likely that a large proportion of the addressees, although formally qualified, never supervised a doctoral thesis project at all. This is reflected by the fact that nearly half (47.3%) of the professors we addressed did respond (252 of 533) as opposed to only 5.3% (6 of 114) of the addressees without a doctor title, but with a university degree such as a master or bachelor.^31^

Overall, the results of the current survey show that, while the vast majority of supervisors are motivated, they feel inadequately prepared for this role. Moreover, from the supervisors' perspective, candidates are not adequately prepared for research and lack both fundamental information and skills for successfully handling the kind of research required for earning the title of medical doctor in Germany.

Some of the deficits thesis supervisors report about in their doctoral candidates are surprising since, following the results of the first survey in 2001,^16^ the Charité graduate programme was specifically initiated and established at the Charité to fill this gap. Initiatives illustrating that the deficits of the system have been recognised also exist at other German medical schools, and similar experiences have been reported.^34–38^ The results of our longitudinal survey among doctoral candidates reflect an improvement in that students consider themselves to be better prepared for scientific work due to obligatory university courses (p<0.001) offered by Charité Medical School.^13^ In addition, participants of the voluntary graduate programme feel significantly better prepared for scientific work (p<0.001). However, the situation is still not optimal with 50% of candidates in 2011 reporting to have received no statistical support during their thesis.^13^ Overall, however, the programme has started to provide some formal training (such as statistics) and has at least to some extent improved the preparation for medical thesis research over the past 14 years.

The deficits supervisors identify among their doctoral candidates are in line with the reports of students that medical studies do not prepare them for research. However, doctoral candidates also state that they expect more assistance from their supervisors with regard to research methodology in general and statistical analysis in particular. This is an interesting aspect as it contradicts the supervisors' perspective—who mostly state that they do provide statistical support. These discrepancies may point to the true core of the problem, namely a failure to communicate and high expectations the other party cannot meet. Lack of a general environment that is conducive to research in terms of providing the necessary infrastructure, resources and time for doing research applies to candidates and supervisors alike: supervisors lack the time to supervise and adequately guide junior medical scientists in doing their first major research project. This is what critics of the German system of medical dissertation deplore, namely that it promotes low scientific standards. Overall, there are two issues to be solved: providing an adequate framework (funding, time, etc), also for supervisors and the basic skills required for adequately planning and managing a scientific project over an extended period of time (from planning to scientific writing).

An improved process quality for collaboratively managing medical thesis projects may improve success rates. Based on our findings, methodological preparation of doctoral candidates, regular training of supervisors in project management and soft skills, and regular meetings of both parties to discuss progress and problems are required and recommended. Once basic information and skills are taught in formal courses, supervisors can spend their time more efficiently by focusing on guiding students in how to apply their theoretical methodological knowledge in conducting their thesis research. In this way, both parties can spend their time more efficiently and candidates will benefit by improving their methodological skill while working on their project.

Our complementary data on candidates' and supervisors' perception of problems in doing doctoral thesis research and completing these projects are important bases for devising strategies to improve the system and help candidates do better research for their doctoral dissertations. Improvements in preparing doctors for supervising medical thesis projects and in preparing medical students for doing research might ultimately also help restore the international reputation of medical dissertations in Germany. On a national level there are many efforts to improve the situation,^8^ ^37^ ^39^ which emphasises that university hospitals have to be structurally adjusted to satisfy the needs of medical research and education.^15^ ^34^ ^35^ ^40^ ^41^ Our findings confirm the idea of adjustment in that universities need to provide the structural framework that is conducive to high-quality research by doctoral candidates.^2^ ^13^ ^16^ ^19^ ^34^

In Europe, there is a debate about a unified medical doctorate. While this is in general a good idea, doing so in the form of a vocational degree might have undesired consequences and plans to abolish the research aspect should be given careful consideration. Clinician scientists are needed in the medical community and would also encourage more consistent application of evidence-based medicine in daily practice throughout the European Union.^15^ ^42^ Formal training of lecturers, tutors and supervisors responsible for this integration would help to standardise and improve the quality of thesis supervision.^17^ ^43^ This could be accomplished by master classes, ‘Teaching the Teachers’,^44^ ^45^ which were implemented to improve the management skills of supervisors at Charité—Universitätsmedizin Berlin.

A graduate programme teaching both doctoral candidates and supervisors to prepare them for their roles in medical thesis research projects and thus helping more candidates in actually completing their thesis projects is recommended. The success of any measures implemented, including a desirable improvement in process quality, can be assessed by repeat surveys.

### Strengths and limitations of this study

To the best of our knowledge, this is the first study that considers the supervisors' perspective on medical thesis projects. Our results are based on a cross-sectional survey and do not reflect temporal dynamics. We conducted a unimodal online survey; therefore the response rate was rather low. Moreover, our survey was conducted at a single university, though one of the largest European medical schools located in Germany and may not be representative of the perspective of medical thesis supervisors elsewhere in Europe.

## Conclusions

We found different perceptions on reasons for constantly high dropout rates of doctoral projects between supervisors and doctoral candidates, which suggest a lack of true scientific collaboration. Based on self-assessments, the vast majority of supervisors (87%) feel inadequately prepared for their supervisory role of medical thesis projects. An improved process quality for collaboratively managing medical thesis projects may improve the chance of success as would a uniform European MD.^11^ ^43^ Master classes preparing medical thesis supervisors for their crucial supervisory role should raise awareness about the differences in perspectives and expectations. This has great potential to foster truly collaborative efforts and a new process quality in the pursuit of medical thesis projects.
